# Clinical outcome of Brazilian patients with non-small cell lung cancer in early stage harboring rare mutations in epidermal growth factor receptor

**DOI:** 10.1590/1414-431X2022e12409

**Published:** 2023-01-09

**Authors:** J. Machado-Rugolo, C.M. Baldavira, T.G. Prieto, E.H.R. Olivieri, A.T. Fabro, C.A. Rainho, E.C. Castelli, P.E.M. Ribolla, A.M. Ab'Saber, T. Takagaki, M.A. Nagai, V.L. Capelozzi

**Affiliations:** 1 Laboratório de Histomorfometria e Genômica Pulmonar, Departamento de Patologia, Faculdade de Medicina, Universidade de São Paulo, São Paulo, SP, Brasil; 2 Centro de Avaliação de Tecnologias em Saúde, Hospital das Clínicas de Botucatu, Faculdade de Medicina, Universidade Estadual Paulista, Botucatu, SP, Brasil; 3 Centro Internacional de Pesquisa/CIPE, AC Camargo Cancer Center, São Paulo, SP, Brasil; 4 Departamento de Patologia e Medicina Legal, Laboratório de Medicina Respiratória, Faculdade de Medicina de Ribeirão Preto, Universidade de São Paulo, Ribeirão Preto, SP, Brasil; 5 Instituto de Biociências, Departamento de Ciências Químicas e Biológicas, Universidade Estadual Paulista, Botucatu, SP, Brasil; 6 Laboratório de Genética Molecular e Bioinformática, Unidade de Pesquisa Experimental, Faculdade de Medicina, Universidade Estadual Paulista, Botucatu, SP, Brasil; 7 Departamento de Patologia, Faculdade de Medicina, Universidade Estadual Paulista, Botucatu, SP, Brasil; 8 Instituto de Biotecnologia, Universidade Estadual Paulista, Botucatu, SP, Brasil; 9 Instituto de Biociências, Departamento de Bioestatística, Biologia Vegetal, Parasitologia e Zoologia, Universidade Estadual Paulista, Botucatu, SP, Brasil; 10 Divisão de Pneumologia, Instituto do Coração, Faculdade de Medicina, Universidade de São Paulo, São Paulo, SP, Brasil; 11 Departamento de Radiologia e Oncologia, Faculdade de Medicina, Universidade de São Paulo, São Paulo, SP, Brasil; 12 Laboratório de Genética Molecular, Centro de Pesquisa Translacional em Oncologia, Instituto do Câncer de São Paulo, São Paulo, SP, Brasil

**Keywords:** Lung cancer, Next generation sequencing, Rare *EGFR* mutation, Platinum-based chemotherapy, Erlotinib

## Abstract

The common epidermal growth factor receptor (EGFR) mutations, such as the L858R point mutation in exon 21 and the in-frame deletional mutation in exon 19, have been definitively associated with response to EGFR-tyrosine kinase inhibitors (EGFR-TKI). However, the clinical outcome and response to treatment for many other rarer mutations are still unclear. In this study, we report the results of Brazilian patients in stage IB-IIIA non-small cell lung cancer (NSCLC) following complete resection with minimal residual disease and *EGFR* mutations treated with adjuvant chemotherapy and/or EGFR-TKIs. The frequency of EGFR mutations was investigated in 70 cases of early stage NSCLC. Mutations in exons 18 and 20, uncommon mutations in exons 19 and 21, as well as in exons 3, 7, 14, 16, 22, 27, and 28, and/or the presence of different mutations in a single tumor (complex mutations) are considered rare. *EGFR* mutations were detected in 23 tumors (32.9%). Fourteen cases carried rare mutations and were treated with platinum-based chemotherapy and two cases were treated with erlotinib. The clinical outcome is described case by case with references to the literature. Notably, we found two rare *EGFR* mutations and one of them with an unknown response to chemotherapy and/or EGFR-TKIs. We have provided complementary information concerning the clinical outcome and treatment of patients with early stage NSCLC for several rare *EGFR* mutations not previously or only rarely reported. Description of cases harboring rare mutations can support the decision-making process in this subset of patients.

## Introduction

In Brazil, the latest 2021 tumor registration data showed that there were approximately 17,760 new cases of lung cancer in men and 12,440 in women (1). Non-small cell lung cancer (NSCLC) accounts for 85% of lung cancer in Brazilian patients (1). Almost 20% of them have early-stage disease (stage I-II) and around 30% of patients have locally advanced disease (stage III) at the time of diagnosis of NSCLC (2). Operative resection is the gold standard curative treatment for patients with NSCLC; nevertheless, local or systemic relapse of the disease is frequent despite complete resection. Five-year overall survival (OS) rates are reported to be 70% for stages IB-IIA, 50-60% for stages IIA-IIB, and 35% for stage IIIA (3). However, some patients experienced a cure for lung cancer by chest surgery alone. In contrast, other patients with completely resected NSCLC may have a minimal residual disease (MRD) not identified radiographically and require adjuvant chemotherapy.

Thus, adjuvant chemotherapy is indicated to avoid recurrence in patients who underwent complete surgical resection for NSCLC. For postoperative patients with stage IIA-IIIA NSCLC, the standard adjuvant treatment is cisplatin-based chemotherapy. Pooled analysis of several clinical trials has shown that adjuvant cisplatin-doublet chemotherapy improved the 5-year disease-free survival (DFS) rate by 5.8% and the 5-year OS rate by 5.4% (4). Moreover, the ADAURA trial demonstrated that osimertinib improves DFS in epidermal growth factor receptor (EGFR) mutation-positive stage IB-IIIA disease following complete resection (5). Therefore, the establishment of the genomic profile and targeted therapies may lead to improvement in therapeutic strategies and the clinical outcomes of lung cancer patients.

The “classical” *EGFR* mutations, such as the L858R point mutation in exon 21 and the in-frame deletional mutation in exon 19, have been undoubtedly associated with target therapy response, while many other EGFR mutations are rarely detected in patients with NSCLC, and information about their association with response to target therapy are still uncertain (6). In cell culture, rare exon 20 mutations make transformed cells less responsive to target therapy, suggesting that other mechanisms probably contribute to primary resistance in metastatic NSCLC (7). For many of the rare mutations, the effect on responsiveness remains unknown. Thus, it is of extreme importance for the clinical decision-making process to share information of patients harboring such mutations, particularly when the outcome of epidermal growth factor receptor-tyrosine kinase receptor (EGFR-TKI) treatment is available.

Previous studies performed by our group using frozen tissue specimens and next-generation sequencing (NGS) from a cohort of 70 patients have shown that Brazilian patients represent a genetically admixed NSCLC cohort with important predictive and prognostic implications (8,9). In the same case series and the array used (QIAamp DNA Mini Kit Qiagen), we detected some Brazilian patients with NSCLC harboring rare *EGFR* variants with clinical significance to be defined.

Therefore, the aim of the present study was to explore the clinical outcome of patients with resected NSCLC in the early stage harboring rare somatic *EGFR* mutations. We also considered mutations in exons 18 and 20, uncommon mutations in exons 19 and 21, and/or the presence of different mutations in a single tumor (complex mutations) as rare mutations. We found 14 cases with rare mutations. These patients were treated with adjuvant chemotherapy and two of them were treated with erlotinib. In this article, we discuss clinical outcomes on a case-by-case basis with references to the literature, if available, or to *in silico* analysis. We also highlight two rare pathogenic mutations that were identified and one case showing EGFR-TKI efficacy with such a mutation. The other rare mutations have been previously described in case reports.

## Material and Methods

### Sample collection

Since novel variants could also arise from sequencing artifacts, especially the ones associated to formalin-related DNA damage as described in Wong et al. (10), our investigation was done in fresh-frozen specimens from Brazilian patients with lung cancer collected during surgical resection from August 2003 to August 2010 at the A.C. Camargo Cancer Center, a tertiary referral center for the treatment of lung cancer in São Paulo, Brazil. The histologic classification was reviewed according to the World Health Organization 2021 classification system (11) and stratified as non-squamous non-small lung cancer (46 cases) and squamous non-small cell lung cancer (n=24). *EGFR* status was associated with baseline characteristics (age, sex, ethnicity, smoking history, stage at diagnosis, histology, type of *EGFR* mutation), treatments (radiotherapy, platinum-based chemotherapy, tyrosine kinase inhibitors), and outcome (relapse-free survival [RFS] after primary surgical resection, relapse, and development of distant metastases).

The study was approved in accordance with the ethical standards of the local committee on human experimentation (Research Ethics Committee of University of São Paulo Medical School - CAAE: 79769017.1.0000.5440; opinion number: 2.673.320). Informed consent was waived because of the retrospective study design. The identity of the subjects under this retrospective analysis was also omitted and anonymized.

### *EGFR* mutation analysis

#### Next-generation sequencing analysis

The DNA of fresh tumor tissue was extracted using the QIAamp DNA Mini Kit (Qiagen, Germany), according to the manufacturer’s recommendations, and quantified using the Qubit^®^ 3.0 Fluorometer (Invitrogen, Life Technologies, USA). The full genomic sequence of EGFR (2 kbp upstream 5’UTR, 2 kbp downstream 3’UTR, coding regions, and intronic sequences) was targeted and captured using TruSeq Custom Amplicon Panel v1.5 kit (TSCAP, Illumina, USA) followed by massively parallel sequencing of enriched fragments on an Illumina MiSeq platform (Illumina) consisting of 150 bp paired-end reads (300 cycles). All tumor specimens had an average sequencing depth of the target region ≥100× and coverage of the target region >90% at 30×.

#### Identification of single nucleotide variants (SNVs) and indels

In order to reduce the effects of PCR amplification and sequencing artifacts, sequencing data analyses were performed at the Molecular Genetics and Bioinformatics Laboratory of the Experimental Research Unit (UNIPEX) at the Medical School of São Paulo State University (UNESP). The raw sequencing data were base-called and demultiplexed using MiSeq Reporter v.1.8.1 (Illumina) with default parameters, and FastQC files were generated for downstream data analysis. Filtered reads were aligned to the human genome (hg19, GRCh37) using the Burrows-Wheeler Alignment tool (BWA; v.0.7.10). After alignment, the SAMtools software (github.com/samtools/samtools) was used to convert the alignment files to an indexed binary alignment map format. The single nucleotide variants (SNVs) and short insertions and deletions (INDELs) were called using the GATK UnifiedGenotyper, including HaplotypeCaller with default parameters based on hg19 and annotated with dbSNP version 144 (https://www.ncbi.nlm.nih.gov/SNP/). To reduce false-positive somatic mutations that might originate from germline variants, the following cut-off criteria were used: number of reads with the altered base in the tumor ≥10, mutations detected at a position of total read depth of ≥100, frequency of the reads with the altered base in the tumor ≥5% except for variants that are also reported in the COSMIC database, minor allele frequency <0.1% in two publicly available databases, namely 1000 Genomes (http://www.1000genomes.org) and Exome Aggregation Consortium (http://exac.broadinstitute.org/).

### *In silico* prediction tools and tumor samples to guide uncommon profiles

The variants were annotated using the VEP software based on the consequences, predicted impacts, and reported allele frequencies in the population. Variants of unknown significance (VUS) were checked on the Cancer Genome Interpreter (https://www.cancergenomeinterpreter.org/home) and VarSome database, a single website that allows access to publications, ClinVar, and all *in silico* prediction tools including the Genomic Evolutionary Rate Profiling (GERP). We also used the Mastermind bioinformatics platform to examine further clinical diagnostic variant interpretation. Next, the mutation plots were generated using the online customized MutationMapper tool at cBioPortal for Cancer Genomics. In this study, all variants were classified according to the American College of Medical Genetics and Genomics (ACMG) recommendations, which allows classification as either “pathogenic”, “likely pathogenic”, “likely benign”, “benign”, or “uncertain significance”.

### Data evaluation

All analyses of the cohort were exploratory. No statistical test was applied given the small size of the cohort, implying a reduced power of significance.

## Results

### Characteristics of the study cohort

Table 1 summarizes the baseline characteristics of the cohort. Seventy Brazilian patients with surgically resected NSCLC were included in the study. The median age of the patients was 78 years (range, 41-96), with similar distribution between older and younger patients. Forty-four patients were male (62.9%), 48 (68.6%) were of European descent, and 21 (30.0%) were smokers. Adenocarcinoma histotype was diagnosed in 46 (65.71%) patients and squamous cell carcinoma in 24 (34.3%) patients. Classification of patients according to TNM staging included 14 individuals (20.0%) in stage IA, 10 individuals (14.3%) in stage IB, 10 individuals (14.3%) in stage IIA, 13 individuals (18.6%) in stage IIB, and 11 individuals (15.7%) in stage IIIA. During the postoperative period, pulmonary and mediastinal relapse occurred in 11 patients (15.7%) and distant metastasis (brain and bone) in 12 patients (17.4%). Of the 70 patients, 23 (32.8%) received platinum-based chemotherapy and two patients (2.9%) received erlotinib. Thirty-eight patients (54.3%) died. The median follow-up was 49 months (range of 0-175). Some cases lacked follow-up information: age [5]; gender [5]; ancestry [18]; smoking status [42]; TNM stage [12]; relapse [34]; and survival status [9].

**Table 1:** Frequency of demographic and clinical characteristics of 70 NSCLC patients.

Characteristics	Number of patients (n=70)
Age, median (range) in years	78 (41-96)
≤77 years	32 (45.7%)
>77 years	33 (47.1%)
Gender	
Male	44 (62.9%)
Female	21 (30.0%)
Ancestry	
European	48 (68.6%)
Asian	2 (2.9%)
African	2 (2.9%)
Smoking status	
Smoker	21 (30.0%)
Non-smoker	7 (10.0%)
Histological subtype	
Adenocarcinoma	46 (65.71%)
Squamous cell carcinoma	24 (34.3%)
Pathological TNM stage^#^	
IA	14 (20.0%)
IB	10 (14.3%)
IIA	10 (14.3%)
IIB	13 (18.6%)
IIIA	11 (15.7%)
Relapse	
No	13 (18.6%)
Local-regional	11 (15.7%)
Distant metastasis	12 (17.4%)
Post-operative treatment	
Chemotherapy (platinum-based)	23 (32.8%)
Radiotherapy	4 (4.3%)
Chemoradiotherapy	6 (8.6%)
Tyrosine kinase inhibitor (erlotinib)	2 (2.9%)
Status for overall survival	
Live	23 (32.9%)
Dead	38 (54.3%)
Follow-up, median (range) in months	49 (0-175)
EGFR status	
Mutation exons 18-21	14 (20.0%)
Uncommon mutation exons 3, 7, 14, 16, 22, 27, 28	9 (12.9%)
Wild type	47 (67.1%)

NSCLC: non-small cell lung cancer. Some cases lacked follow-up information: age (5); gender (5); ancestry (18); smoking status (42); TNM stage (12); relapse (34); and survival status (9). ^#^International Association for the Study of Lung Cancer (8th edition).

### Frequency of *EGFR* somatic mutations

**Figure 1:**
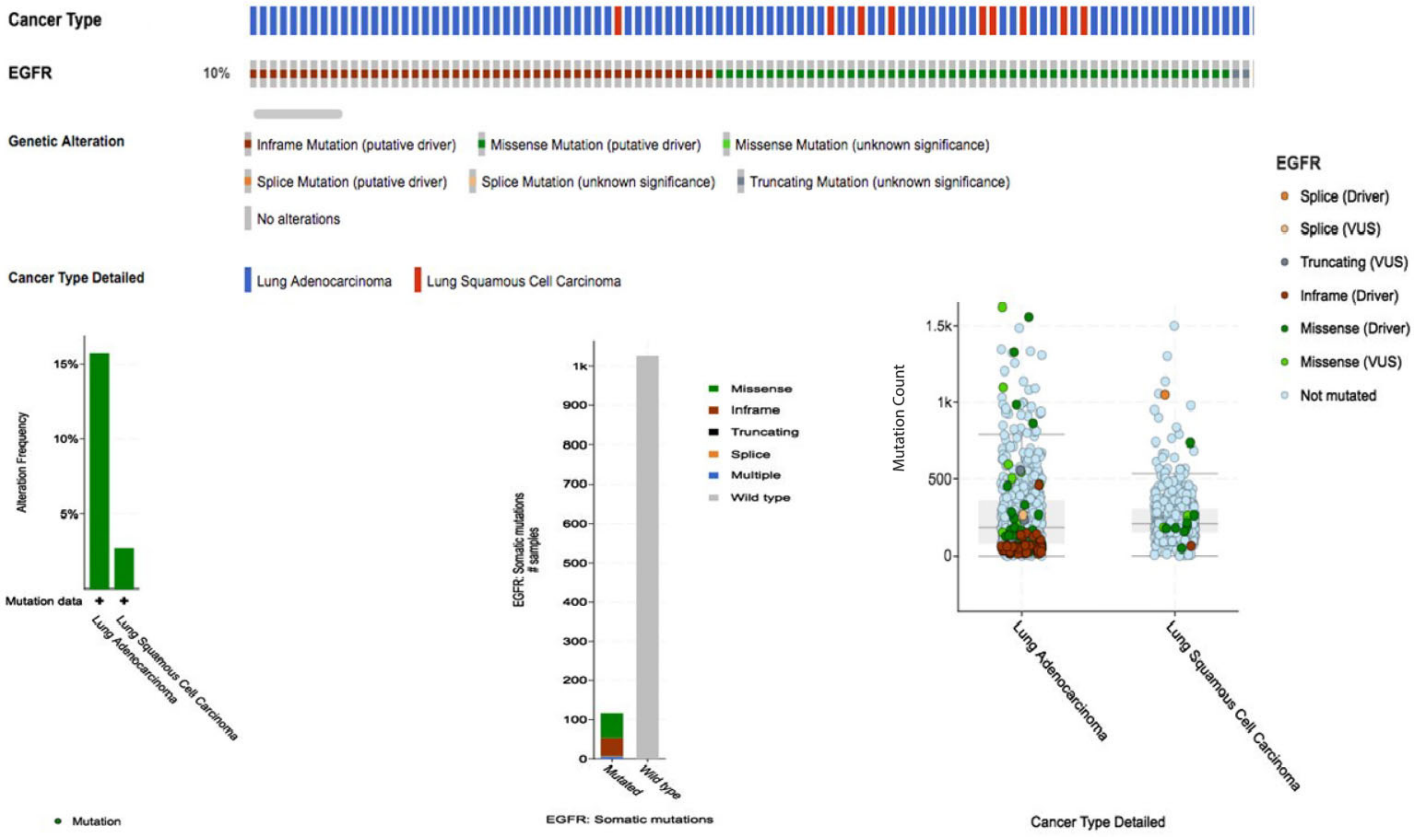
The *EGFR* gene in lung cancer from The Cancer Genome Atlas (TCGA) database. The Oncoprinter showed the distribution and type of *EGFR* gene mutations in lung cancer samples (https://www.cbioportal.org/). VUS: Variants of unknown significance.

In our cohort, *EGFR* mutations (target exons 1-28) were detected in 23 of the 70 patients (32.9%), 44 males and 21 females. Classic or sensitizing activating mutations (exons 18-21) occurred in 14 patients (20.0%), being 9 of 14 (64.2%) in exon 19 (in-frame deletion). Uncommon mutations in exons 3, 7, 14, 16, 22, 27, and 28, and/or complex mutations were identified in 9 tumors corresponding to 12.9% of the entire population analyzed. The results of NGS sequences are listed in detail in Tables 2 and 3. Figure 1 shows an illustration obtained from cBioPortal jargon and bar plots with data of mutation frequency, somatic mutation variants, and mutation code.

**Table 2:** Spectrum of *EGFR* variants in exons 18-21 identified in a Brazilian NSCLC cohort by Next-Generation Sequencing.

Exon/Intron	ID rs	HGVS nucleotide	HGVS protein	Variant type	Frequency	ACMG classification
18	rs28929495	c.2155G>T	G719C (p.Gly719Cys)	Missense	1	Pathogenic
19	rs121913229	c.2248G>C	A750P (p.Ala750Pro)	Missense	7	Likely pathogenic
19	rs121913421	c.2235_2249del	E746_A750del (p.Glu746_Ala750del)	inframe_deletion	5	Pathogenic
19	rs121913436	c.2239_2247delTTAAGAGAA	L747_E749del (p.Leu747_Glu749del)	inframe_deletion	1	Likely pathogenic
19	rs121913442	c.2240_2254del	L747_T751del (p.Leu747_Thr751del)	inframe_deletion	1	Likely pathogenic
20	rs121913465	c.2303G>T	S768I (p.Ser768Ile)	Missense	1	Pathogenic
20	rs121434568	c.2438T>G	L813R (p.Leu813Arg)	Missense	2	Pathogenic
20	rs1275022697	c.2326C>T	R776C (p.Arg776Cys)	Missense	1	Likely pathogenic
21	rs148934350	c.2543C>T	P848L (p.Pro848Leu)	Missense	1	Pathogenic

EGFR: epidermal growth factor receptor; NSCLC: non-small cell lung cancer; rs: reference single nucleotide polymorphism; HGVS: Human Genome Variant Society; ACMG: American College of Medical Genetics and Genomics.

**Table 3:** Spectrum of uncommon *EGFR* variants identified in a Brazilian NSCLC cohort by Next-Generation Sequencing.

Exon/Intron	ID rs	HGVS nucleotide	HGVS protein	Variant type	Frequency	ACMG classification
3	rs754854319	c.409A>G	M137V (p.Met137Val)	Missense	3	Uncertain significance
7	rs149840192	c.866C>T	A289V (p.Ala289Val)	Missense	1	Likely pathogenic
8	rs886037891	c.977G>T	C326F (p.Cys326Phe)	Missense	1	Likely pathogenic
14	rs144943614	c.1639G>A	V547I (p.Val547Ile)	Missense	1	Uncertain significance
14	rs779076899	c.1705G>A	G569S (p.Gly569Ser)	Missense	1	Uncertain significance
15	rs538888597	c.1881-462C>T	*	Intron	1	Uncertain significance
16	rs369399038	c.1903C>T	R635W (p.Arg635Trp)	Missense	1	Uncertain significance
22	rs376822837	c.2611G>A	D871N (p.Asp871Asn)	Missense	1	Uncertain significance
27	rs35918369	c.3494C>T	A1165V (p.Ala1165Val)	Missense	1	Uncertain significance
28	rs869064669	c.*582T>G	*	3_prime_UTR_variant	1	Uncertain significance
28	rs751311059	c.*708T>C	*	3_prime_UTR_variant	1	Uncertain significance
28	rs988454507	c.*934G>A	*	3_prime_UTR_variant	1	Uncertain significance
28	rs771422383	c.*1060G>A	*	3_prime_UTR_variant	1	Uncertain significance

EGFR: epidermal growth factor receptor; NSCLC: non-small cell lung cancer; rs: reference single nucleotide polymorphism; HGVS: Human Genome Variant Society; ACMG: American College of Medical Genetics and Genomics. The asterisks indicate lack of information.

### Clinical outcome in patients harboring mutation clusters in exons 18-21

#### Exon 18 mutations - G719C (exon 18) + S768I (exon 20)

Patient report: The G719C + S7681 pathogenic double mutation was found in a 70-year-old male smoker of European ancestry and with adenocarcinoma showing micropapillary characteristics in stage IIIA. After local recurrence, the patient was treated with a combination chemotherapy of CDDP and etoposide. The patient died 13 months after the surgical resection. Reference: The complex mutation has been previously described in a few reports (12,13). *In vitro* data suggest that the coexistence of the E709A + G719C mutation confers resistance to adjuvant treatment, including EGFR-TKIs (14). Another report did not confirm the low sensitivity to adjuvant treatment of the double mutant (15). Comment: According to the cases described so far, the *G719C + S768I* complex mutation seems to be associated with resistance to adjuvant treatment including EGFR-TKI.

#### Exon 19 mutations - p.E746_R748del/p.A750P

Patient report: The p.E746_R748del/p.A750P pathogenic complex mutation was found in two nonsmokers female patients, a 90-year-old and 96-year-old, both of European descent, and with adenocarcinoma stage IIIA with acinar and solid characteristics. After pulmonary and mediastinal local relapse, both patients were treated with carboplatin and Taxol, which was interrupted early due to toxicity. A clinically meaningful improvement of symptoms was obtained within a few days and a partial response of tumor assessment was obtained after 6 weeks. After 9 months, both patients presented disease progression and died. Reference: The presence of p.E746_R748del/p.A750P has already been reported in one Caucasian patient by Jakobsen et al. (16) but without specific information on the efficacy of EGFR-TKI. Comment: Similar to the single case reported in the literature showing progression following gefitinib, the two patients treated in our institution had a rapid symptomatic improvement but died 9 months after disease progression.

#### Exon 20 mutations - L813R + R776C

Patient report: The L813R + R776C EGFR mutation was found in a 70-year-old male, current heavy smoker, of European descent, and with adenocarcinoma stage IIIA with acinar characteristics. After pulmonary local relapse, the patient was treated with CDDP/Navelbine. Tumor progression was observed after 10 weeks, at the first treatment assessment. Erlotinib was subsequently given at the starting dose of 150 mg daily. The patient experienced 30 months of progression-free survival. Reference: The *L813R + R776C* mutation was previously described by Han et al. (17) in a patient with squamous NSCLC, who benefited from afatinib and experienced 11 months of progression-free survival. The same mutation has been found by Moran et al. (18), but in association with the L861 in exon 20, in a never-smoking patient with adenocarcinoma with lepidic features. In this case, the patient had a complete and long-lasting remission after afatinib treatment. Comment: Similar to the case reported by Moran where the L861 was linked to *R776C*, in our experience the *L813R + R776C* EGFR mutation was associated with response to erlotinib.

#### Exon 21 mutations - P848L

Patient report: The P848L mutation was found in a 94-year-old male, current heavy smoker, of European descent, and with squamous cell carcinoma with non-keratinizing features. After lobectomy, the tumor pathological stage was classified as IB. During the first three months of follow-up, the patient developed pulmonary relapse and bone metastases. He was treated with radiotherapy and a combination of CDDP/Navelbine, followed by erlotinib with rapid tumor progression, and died. Reference: P848L has been described in 16 carcinoma patients in the Catalogue of Somatic Mutations in Cancer (COSMIC) (19), accounting for about 0.6% of cancer patients (20). A recent study showed that the P848L germline variant of EGFR contributes to cellular transformation and predicts resistance to TKIs, and that patients harboring P848L likely benefits from JAK inhibitors (21). Furthermore, very rare exon 21 mutations have shown to be more sensitive to afatinib (second-generation EGFR-TKIs) and osimertinib (third-generation EGFR-TKIs) than first-generation EGFR-TKIs in preclinical studies (22). Comment: This is an unusual case of a patient with a tumor in stage IB harboring a P848L mutation with no response to adjuvant treatment, including TKI, and rapid progression of the disease.

### Clinical outcome of patients harboring rare *EGFR* mutations with uncertain significance

#### Exon 3 mutations - M137V

Patient report: M137V mutation was found concurrently with other two unknown mutations, c.1881-462C>T (intron 15) and A1165V (exon 27), in a 78-year-old non-smoker female of European descent and with adenocarcinoma showing lepidic characteristics. The patient underwent pneumectomy and the tumor was staged as IB. She received adjuvant radiotherapy and a combination of CDDP with Navelbine with partial response of the tumor and is alive after 8 years of follow-up. Reference: The M137V mutation and the c.1881-462C>T and A1165V variants are reported in the dbSNP database (rs754854319) without any data regarding their clinical significance. Chakroborty et al. (23) described this alteration in colon adenocarcinomas from human samples available in the GENIE database. VarSome database predicted their significance to be uncertain. The DEOGEN2, FATHMM-MKL, LIST-S2, M-CAP, MutationAssessor, MutationTaster, and SIFT computational analyses inferred pathogenicity, while BayesDel_addAF, DANN, EIGEN, MVP, and PrimateAI tools) predicted benignity. The GERP score (5.13) indicates that the Met amino acid in position 137 lies in a highly conserved region, which also supports the prediction of pathogenicity. Comment: Our patient with adenocarcinoma stage IB harboring M137V mutation responded partially to adjuvant treatment and is alive after 8 years of follow-up.

#### Exon 7 mutations - A289V

Patient report: The A289V mutation was found in a 94-year-old male patient of European descent and with adenocarcinoma with solid characteristics staged as IIB. The patient died two months after surgical resection. Reference: The A289V mutation in exon 7 encodes an amino acid of the extracellular domain of the EGFR protein and has been found only in the glioblastoma, low-grade glioma, head, and neck neoplasms, being rarely described in NSCLCs (24,25). Dai et al. (25) were the first to present a case report of NSCLC in a Chinese patient with *EGFR* p.A289V mutation, whose treatment showed only a partial response to first-generation EGFR-TKIs. The A289V mutation was classified as likely pathogenic by ACMG. VarSome database analysis showed a pathogenic computational result based on ten pathogenic predictions from BayesDel_addAF, DANN, DEOGEN2, EIGEN, FATHMM-MKL, LIST-S2, M-CAP, MutationAssessor, MutationTaster, and SIFT *vs* two benign predictions from MVP and PrimateAI. Comment: The A289V mutation seems to occur in patients with advanced age in the early stage of the disease rapidly progressing to death.

#### Exon 8 mutations - C326F

Patient report: C326F mutation was detected in a stage IIB keratinizing squamous cell carcinoma from a 89-year-old smoker patient who underwent pneumonectomy and died one month later. Reference: This genomic alteration has been described in a case report of Lhermitte-Duclos disease, also known as dysplastic cerebellar gangliocytoma, which has been shown to be an activation mutation resulting in increased *EGFR* autophosphorylation and indicating high receptor activation (26). VarSome's *in silico* analysis of the C326 mutation revealed 11 pathogenic predictions from BayesDel_addAF, DANN, DEOGEN2, EIGEN, FATHMM-MKL, LIST-S2, M-CAP, MVP, MutationAssessor, MutationTaster, and SIFT *vs* one benign prediction from PrimateAI. Comment: The C326F also occurred in a patient with advanced age with squamous cell carcinoma in an early stage disease rapidly progressing to death.

#### Exon 14 mutations - V547I-G569S

Patient report: The V5471-G569S mutation, concomitantly with a point mutation R776C, was found in a 69-year-old male patient of African descent and with non-keratinizing squamous cell carcinoma, stage IIB, who presented pulmonary relapse 11 months after surgical resection. The patient was treated with radiotherapy and a combination of carboplatin and Taxol but died due to brain metastases. Reference: VarSome database analysis revealed eight pathogenic predictions for the V5471-G569S mutation according to DANN, EIGEN, EIGEN PC, FATHMM-MKL, FATHMM-XF, LRT, MutationAssessor, and MutationTaster against 11 benign predictions from BayesDel addAF, BayesDel noAF, DEOGEN2, FATHMM, LIST-S2, MVP, MetaLR, MetaSVM, PrimateAI, REVEL, and SHIFT 4G. The G569S mutation with concomitant point mutation R776C in the VarSome computational analysis led to a benign computational result based on 12 benign predictions from BayesDel_addAF, DANN, DEOGEN2, EIGEN, FATHMM-MKL, LIST-S2, M-CAP, MVP, MutationAssessor, MutationTaster, PrimateAI, and SIFT *vs* no pathogenic predictions. Comment: In our case, the V5471-G569S mutations were detected in non-keratinizing squamous cell carcinoma, stage IIB, from a patient of African ancestry who did not respond to adjuvant chemotherapy and died after brain metastases.

#### Exon 16 mutations - R635W

Patient report: The mutation R635W was found in a 64-year-old non-smoker female patient with adenocarcinoma, stage IIA, who received adjuvant chemotherapy and radiotherapy and died three years after the surgery. Reference: The mutation R635W is reported in the dbSNP database (rs369399038) of uncertain significance. *In silico* analysis predicted a deleterious effect based on 11 pathogenic predictions from BayesDel_addAF, DANN, DEOGEN2, EIGEN, FATHMM-MKL, LIST-S2, M-CAP, MutationAssessor, MutationTaster, PrimateAI, and SIFT *vs* one benign prediction from MVP. Comment: Another patient from our institution with stage IIA adenocarcinoma harboring the mutation R635W experienced partial response to radiotherapy and chemotherapy with rapid progression of the disease.

#### Exon 22 mutations - D871N

Patient report: The D871N mutation was found in a 69-year-old heavy smoker male patient of African ancestry and with keratinizing squamous cell carcinoma, stage IIB, who underwent pneumectomy. The tumor relapsed in the central nervous system. The patient received a combination of carboplatin and Taxol and died eleven months later. Reference: The VarSome database produced a final likely pathogenic computational result based on seven pathogenic predictions from DANN, DEOGEN2, FATHMM-MKL, LIST-S2, M-CAP, MutationTaster, and SIFT *vs* five benign predictions from BayesDel_addAF, EIGEN, MVP, MutationAssessor, and PrimateAI. Comment: Another uncommon mutation harbored in squamous cell carcinoma from a patient with poor tumor response.

#### Exon 28 and Exon 29 mutations - Non-coding

Patient report: Three male patients older than 70 years with IIA adenocarcinoma harboring c.*582T>G (rs869064669), c.*708T>C (rs751311059), c.*934G>A (rs988454507), and c.*1060G>A (rs771422383) who underwent lobectomy and received a combination of CDDP with Navelbine were alive nine years after surgical resection. Reference: The four VarSome analyses resulted in benign computational results, with one benign prediction from DANN *vs* no pathogenic prediction in all four cases. Moreover, all four variants had a GERP score of less than 5.5, an indication that the position of these variants was not highly conserved (-0.878, 3.56, -2.5, and -0.396, respectively). Comment: It is worth noting, however, that the use of more sensitive sequencing techniques might reveal rare mutants at a higher frequency.

## Discussion

Previous studies from the present case series have shown that Brazilian patients represent a genetically admixed NSCLC cohort with clinical implications. In fact, we found that patients with adenocarcinoma harboring somatic mutations of *EGFR*, *CTLA4*, *PDCD1LG2*, or *ZEB2* that only underwent surgical treatment and developed brain metastases may have the worst prognosis (8,9). *EGFR* somatic mutations were detected in approximately 13% of NSCLC Brazilian patients with clinical significance to be defined. These findings motivated us to re-analyze *EGFR* mutations in the 70 case series of NSCLC. Because of the small sample size, we discussed their clinical outcome case by case with references to the literature, and not based on power for significance.

Under the objectives of the present study, we found: 1) rare mutations, uncommon mutations, and/or complex somatic mutations identified in 20% of the Brazilian sample analyzed, who were mostly of European ancestry, non-smoker males older than 77 years, with adenocarcinoma histology stage IB-IIB; 2) rare *G719C* (*exon 18*) *+ S768I* (*exon 20*) complex somatic mutation seems to be associated with resistance to combination chemotherapy of CDDP and etoposide with short progression-free survival (PFS); 3) rare *p.E746_R748del/p.A750P* complex somatic mutations resistant to carboplatin and Taxol with short PFS; 4) rare *L813R + R776C* (*exon 20*) complex somatic mutation associated with response to erlotinib and prolonged PFS; 5) rare *P848L* (*exon 21*) somatic mutation resistant to combination of CDDP/Navelbine and erlotinib with short PFS; 6) uncommon *M137V* (*exon 3*) and non-coding (*exons 28 and 29*) somatic mutations with response to combination of CDDP with Navelbine and prolonged PFS; 7) uncommon somatic mutations *A289V* (*exon 7*), *C326F* (*exon 8*), *V547I-G569S* (*exon 14*), *R635W* (*exon 16*), and *D871N* (*exon 22*) with resistance to the combination of carboplatin and Taxol and short PFS.

The frequency of *EGFR* mutations in NSCLC range from 5 to 20%, depending on the cohort studied (6). Erlotinib and gefitinib are small molecules that target *EGFR* tumor mutation and are effective in the treatment of patients with this subgroup of tumors, showing a response rate of approximately 70% (27). Two classical activating mutations represent the most common mutations: a short in-frame deletion of exon 19 and a point mutation (CTG to CGG) in exon 21 at nucleotide 2573 leading to the replacement of leucine by arginine at codon 858 (L858R). However, other *EGFR* mutations do not effectively cause *EGFR* tyrosine-kinase activity and may be associated with acquired TKIs resistance, therefore affecting the sensitivity of the EGFR-TKIs (28). In the case of rare EGFR mutations, the literature is very limited regarding clinical outcomes and response to treatment and may underpower the value of target therapy.

Therefore, from the *EGFR* mutation screening performed on our patients, we detected NSCLC with rare *EGFR* mutations and showed the response rate after chemotherapy and erlotinib treatment. In detail, we analyzed 70 tumors and identified 14 cases with rare mutations, defined as mutations in exons 18, 20, and 21, uncommon mutations in exons 3, 7, 14, 16, 22, 27, 28, and/or complex mutations (different mutations present in a single tumor). These mutations accounted for approximately 20% of all the screened cases coinciding with other studies in South America including Brazil (29) and Colombia (30), but lower than rates found in Asia and higher than rates of European populations (31,32). The current cohort included patients from all regions of the country who came to a tertiary referral center for the treatment of lung cancer. These patients present different education levels, socioeconomic status, and ancestry characterizing a genetic admixture, inherited from European, African, and Asian immigrants (31,32). As expected, we observed a high prevalence of rare and uncommon *EGFR* mutation in Brazilian patients with European ancestry compared to those found in the native European population (33). Twenty-three of our patients were treated with platinum-based chemotherapy and two patients received erlotinib, and we reported the individual case reports regarding the literature and *in silico* analysis. We observed that patients with rare *EGFR* mutations could receive platinum-based chemotherapy as a first-line treatment, due to their low response rates and short PFS in response to erlotinib. The clinical outcome supported the different predictive values of the single *EGFR* mutations in terms of treatment effectiveness. Of note, in our cohort, exon 20 mutations were associated with erlotinib sensitivity, and the patient experienced 30 months of PFS; however, there are only a few reports of cases responding to EGFR-TKIs in the literature. Therefore, we inferred that the evaluation of the single mutations, case by case, could be useful also in the presence of exon 20 mutations, as in the presence of other uncommon *EGFR* mutations.

Although we showed the potential of NGS technique in a small cohort of NSCLC, future validation is necessary using a similar cohort with a large set of patients to corroborate the results observed in this tumor tissue type. The main limitation of our exploratory analysis was the small number of NSCLC cases used, but this was minimized by the data obtained using this technique. The present study was largely descriptive and exploratory, and extension of our findings is essential. Another limitation of our research was that the comprehensive interpretation of sequence variants requires not only well-established databases but also appropriate functional analyses, such as evaluating the effect of gene expression of these detected variants.

In summary, we have provided complementary information concerning the effectiveness of chemotherapy and erlotinib in patients with early stage NSCLC for several rare *EGFR* mutations not previously or only rarely reported. These data together with similar studies in the literature and *in silico* analysis reinforce the decision-making process in such subsets of patients.
